# Expressions of fatty acid synthase and HER2 are correlated with poor prognosis of ovarian cancer

**DOI:** 10.1007/s12032-014-0391-z

**Published:** 2014-11-30

**Authors:** Yunlang Cai, Jingmei Wang, Lin Zhang, Di Wu, Dandan Yu, Xiaoqiang Tian, Jun Liu, Xinru Jiang, Yang Shen, Lihua Zhang, Mulan Ren, Peilin Huang

**Affiliations:** 1 Department of Obestetrics and Gynecology, Zhongda Hospital, Southeast University, Dingjia Qiao Road 87, Nanjing, 210009 Jiangsu Province China; 2 Department of Pathology, Drum Tower Hospital, Nanjing, 210009 Jiangsu Province China; 3 Medical College, Southeast University, Dingjia Qiao Road 87, Nanjing, 210009 Jiangsu Province China; 4 The Second Hospital of Nanjing, Zhongfu Road 1, Nanjing, 210003 Jiangsu Province China; 5 Department of Pathology, Zhongda Hospital, Southeast University, Dingjia Qiao Road 87, Nanjing, 210009 Jiangsu Province China

**Keywords:** Fatty acid synthase, HER2, Ovarian cancer, Clinicopathological factors

## Abstract

The present study was designed to explore the cross talk between fatty acid synthase (FASN) and HER2 (ErbB2) in ovarian cancer. A total of 60 ovarian cancer patients and 15 normal ovarian tissues were enrolled. Tissue array was conducted by using a tissue microarray instrument. Immunohistochemistry was performed to quantify the expressions of HER2 and FASN. The FASN was detected to be distributed in the cell cytoplasm and was significantly correlated with cancer grade (*p* = 0.000) and FIGO staging (*p* = 0.000). Patients with FASN overexpression in ovarian cancer tend to have a worse overall survival rate (*p* = 0.000). HER2 was also stained to be distributed in the cell cytoplasm associated with higher expression in high-grade cancer. It was also disclosed that FASN expression level is not correlated with HER2 status in ovarian cancer. These results for the first time indicated that a cross talk in FASN and HER2 expressions might be associated with prognosis in malignant ovarian cancer.

## Introduction

Fatty acid synthase (FASN), one of the most important biosynthetic enzymes in lipogenesis, is regulated by hormones, growth factors and diet. Little or no FASN expression is disclosed to be in normal cells and tissues except for liver and adipose tissue [1]. However, FASN expression is often upregulated in rapid proliferation cells. Inhibition of FASN expression could repress cell proliferation in various cancers [2]. For those reasons, FASN has become as an attractive target for cancer therapy in last 15 years [3].

HER2 (ErbB2) is a member of the epidermal growth factor receptor (EGFR) family of receptor tyrosine kinases (RTKs) that play a pivotal role in oncobiological processes [4]. The ‘‘HER2–PI3K/Akt–FASN axis’’ is involved in the regulation of malignant phenotype in colorectal cancer cells [5]. It is reported that a cross talk between ErbB and FASN mediates ovarian cancer cells proliferation [6]. Mounting evidences indicate that FASN gene network and HER2 oncogene system have synergetic effect in tumorigenesis. We speculated that whether an ErbB/FASN cross talk plays a vital role in mediating malignant phenotype of ovarian cancer. Tissue array and immunohistochemistry were used for analysis of FASN and HER2 expressions, and the correlations of FASN and HER2 expressions with clinicopathological factors were statistically analyzed.

## Materials and methods

### Patients

This study was approved by the Ethics Committee of ZhongDa Hospital, and the informed consent was obtained from all subjects. All experiments were performed in accordance with relevant guidelines and regulations. Patients were treated in the ZhongDa Hospital between 1997 and 2000. The study population is consisted of 60 ovarian cancer patients; 15 normal ovarian tissues were used for control group. The summary of patient’s characteristics is shown in (Table 1).

**Table 1:** Association between FASN and HER2 expressions and clinicopathological factors in patients with ovarian clear cell carcinoma

Factors	Patients	FASN			HER2		
		High	Low		High	Low	
Histological type							
Serous	64	49	15	*p* = 0.06	20	44	*p* = 0.372
Mucinous	14	10	4		7	7	
Endometrioid	17	8	9		5	12	
Cancer grade							
G1	21	10	16	*p* = 0.000	8	13	*p* = 0.887
G2	12	5	7		4	8	
G3	62	52	10		20	42	
FIGO stage							
I	8	1	7	*p* = 0.000	1	7	*p* = 0.204
II	10	3	7		3	7	
III	60	45	15		19	41	
IV	17	16	1		9	8	
Residual tumor							
<1 cm	68						
≥1 cm	27						
Age (years)							
<50	38						
≥50	57						
HER2							
High	32						
Low	63						

### Tissue array

Tumor tissues were obtained at the first laparotomy with no neoadjuvant chemotherapy and any other treatment. Slides stained with hematoxylin and eosin (H–E) were generated from the original paraffin blocks that were analyzed for the diagnosis of epithelial ovarian cancer (EOC). Tissue microarray (TMA) was designed after selection of the most representative areas by pathologist. For each block, triplicate 0.8 mm cores of tumor were placed on a TMA, which was performed by Dr. Wangjinei (Drum Tower Hospital, Nanjing, China) using a TMA instrument.

### Immunohistochemistry

The tissue array was hydrated in gradient alcohol, and antigen retrieval was performed in ethylenediaminetetraacetic acid (EDTA)-containing antigen retrieval buffer (pH = 8.0) in 95 °C followed by 3 % H_2_O_2_ incubation for 30 min. After blocking by goat serum for 10 min, mouse anti-human FASN monoclonal antibody and mouse anti-human HER2 monoclonal antibody were incubated overnight at 4 °C. The corresponding Horseradish peroxidase (HRP)-conjugated secondary antibodies were incubated for 30 min at room temperature before visualization by diaminobenzidine (DAB) reagent. The tissue array slides then were stained with hematoxylin and mounted with slide cover for microscopic evaluation. FASN and HER2 staining intensity were scored independently by two observers. Immunoreactivity was scored as follows: 0 (undetectable), + (weakly positive), ++ (moderately positive), +++ (intensely positive).

### Statistical analyses

The data were statistically analyzed with SPSS 16.0 software. The correlation analysis was performed with Spearman’s test. *p* < 0.05 was considered as statistically significant.

## Results

### Relationship between FASN expression and clinicopathological factor in ovarian cancer

**Fig. 1:**
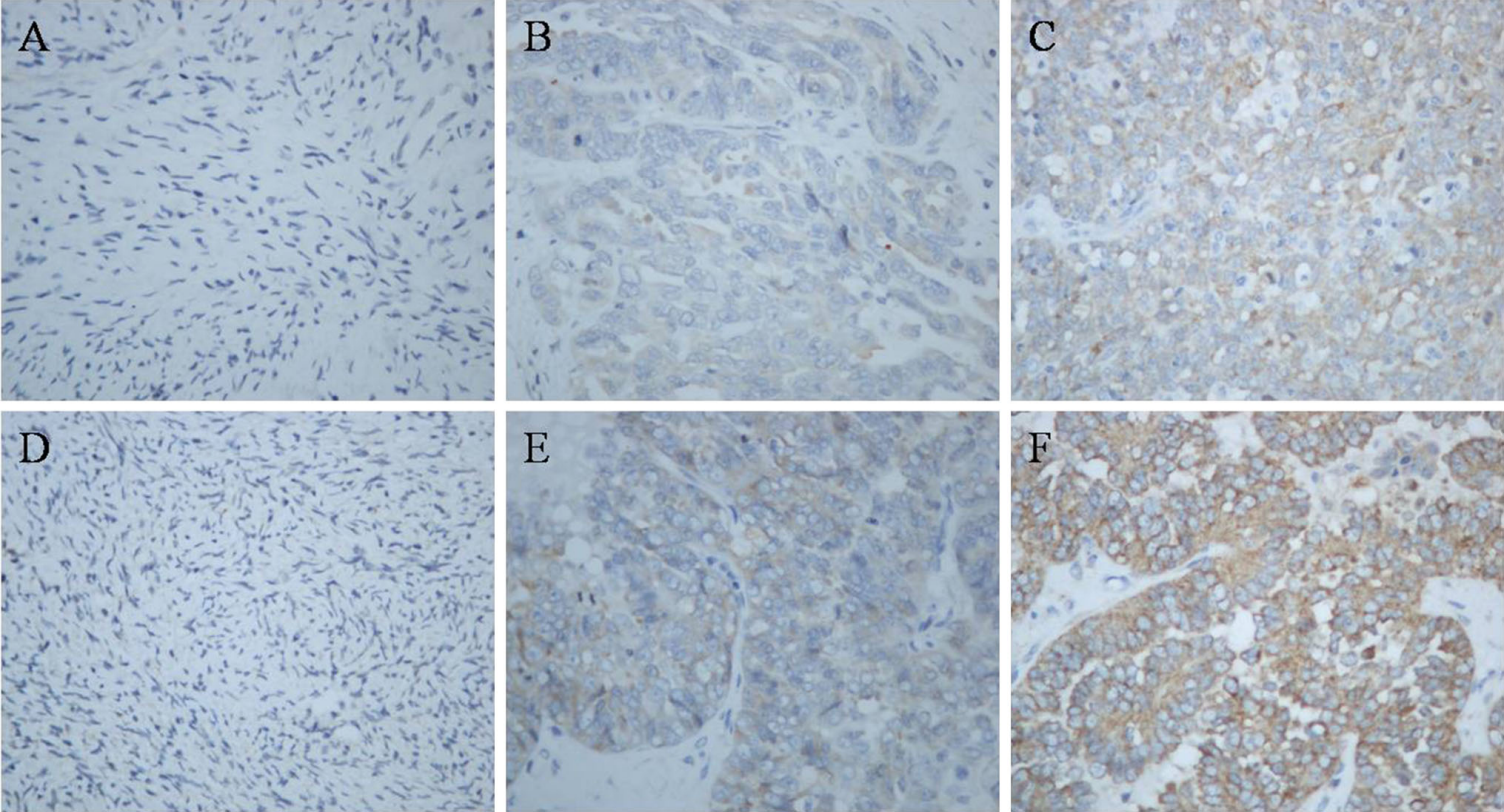
Immunostaining showing regional diversity of FASN and HER2 expressions in ovarian tissues and ovarian cancers. Immunostaining showing regional diversity of FASN/HER2 expression in ovarian tissues (**a**/**d**). The expression levels were graded in a 4-point scale. 0 (undetectable), + (weakly positive), ++ (moderately positive), and +++ (intensely positive); Low (0 and +) and high (++ and +++) expressions of FASN in tissue samples (**b**, **c**). Low (0 and +) and high (++ and +++) expressions of HER2 in tissue samples (**e**, **f**)

**Fig. 2:**
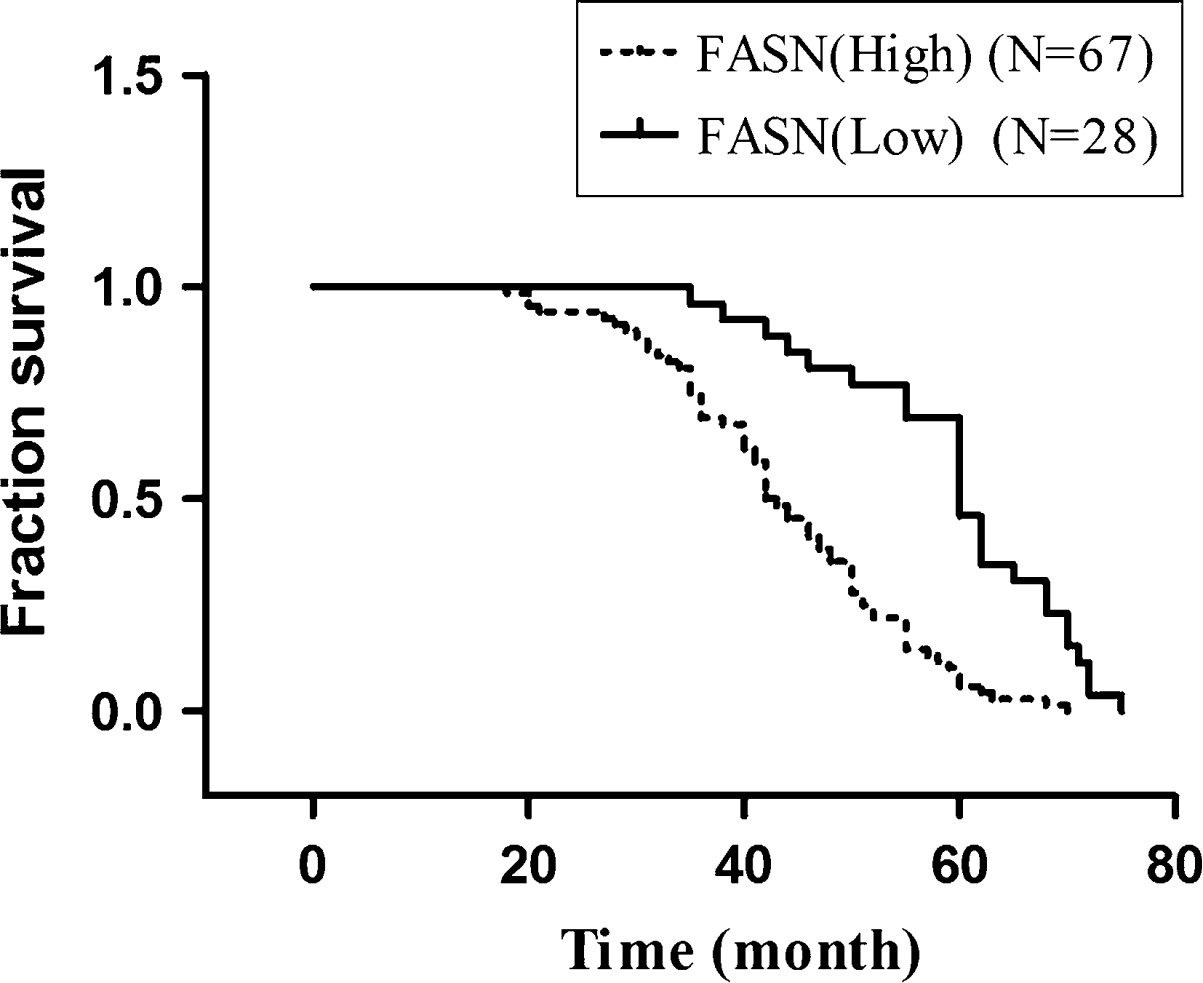
Overall survival based on the expression of FASN. Kaplan–Meier survival analysis showing that high expression of FASN (++ and +++) was associated with a shorter overall survival in comparison with low FASN expression (0 and +) in ovarian cancer (*p* = 0.000, log-rank test)

FASN was detected to be distributed in the cell cytoplasm and high FASN expression levels were observed in 70.5 % (67/95) of analyzed tumors. There was no FASN immunoreactivity (0/15) in the normal ovarian tissues (Fig. 1). Depending on FASN immunostaining score, patients were divided into high expression (2+ and 3+) and low expression (− and +) groups. There was no significant association between FASN status and histological type (*p* = 0.06), patients age (*p* = 0.650), residual tumor (*p* = 0.455). However, cancer grade (*p* = 0.000) and FIGO staging (*p* = 0.000) were significantly correlated with FASN expression (Table 1). Patients with FASN overexpression in ovarian cancer tend to have a worse overall survival rate (*p* = 0.000) (Fig. 2).

### Relationship between HER2 expression and clinicopathological factor in ovarian cancer

**Fig. 3:**
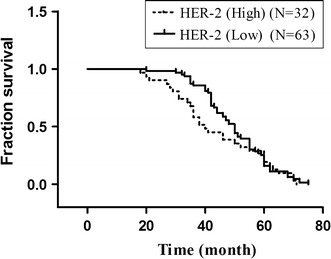
Overall survival based on the expression of HER2. Kaplan–Meier survival analysis showing that high expression of HER2 (++ and +++) was not associated with overall survival in comparison with low HER2 expression (0 and +) in ovarian cancer (*p* = 0.272, log-rank test)

In the pathological section, HER2 was also stained to be distributed in the cell cytoplasm (Fig. 1). Only one normal ovarian tissue shows HER2 positive results (+) (1/15). There was no significant association between HER2 status and patient age (*p* = 0.826), residual tumor (*p* = 0.471), histological type (*p* = 0.372), cancer grade (*p* = 0.887) and FIGO staging (*p* = 0.077). HER2 has more intensity expression in high-grade cancer (Table 1). However, no statistically significance with respect to all tumors (*p* = 0.077) (Fig. 3).

### FASN expression level is not correlated with HER2 status in ovarian cancer

**Fig. 4:**
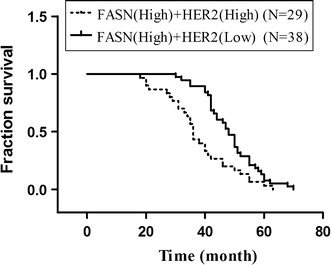
Overall survival based on the expression of FASN and HER2. To confirm the finding that FASN and HER2 expressions were correlated in ovarian cancer, patients were divided into high expression of FASH (high)/HER2 (high) and FASN (high)/HER2 (low) group. Kaplan–Meier survival analysis showing that FASH (high)/HER2 (high) group was associated with a shorter overall survival in comparison with low FASN (high)/HER2 (low) group in ovarian cancer (*p* = 0.002, log-rank test)

**Fig. 5:**
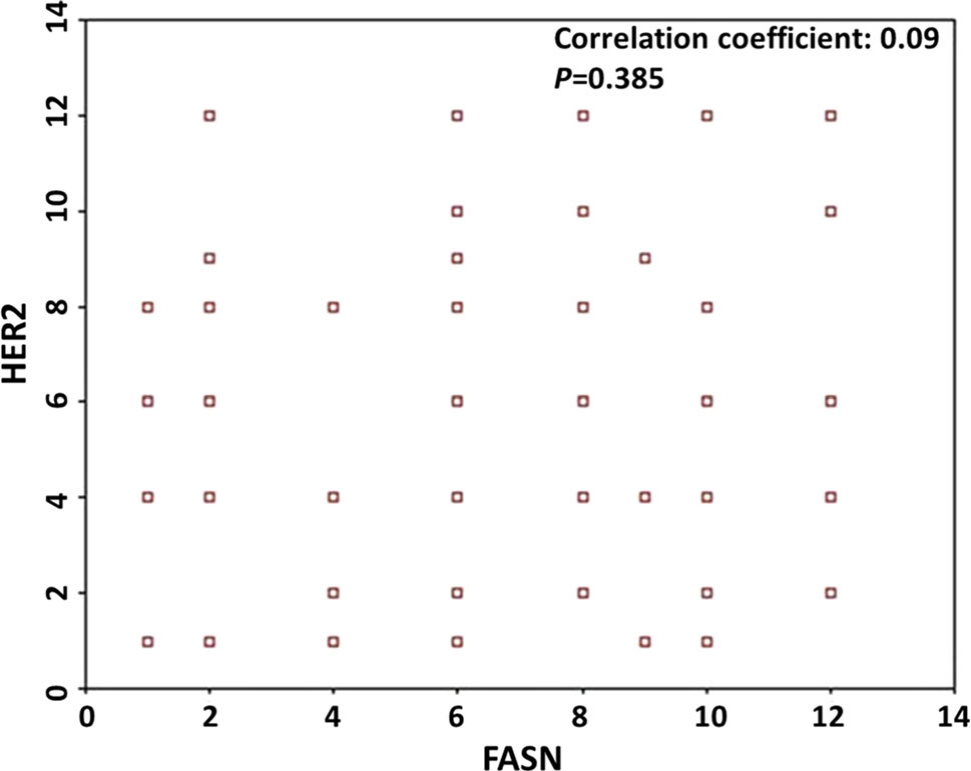
Correlation of expression FASN and HER2 in ovarian cancer. To investigate the expression correlation of FASN and HER2 in ovarian cancer, Spearman correlation analysis showed that FASN and HER2 expressions have no correlation in ovarian cancer (*p* = 0.385, CC: 0.09)

We analyzed the relationship between FASN and HER2 expressions. According to ICH scores in tissues array, there was no significant correlation in ovarian cancer (*p* = 0.385). Nevertheless, to confirm the finding that FASN and HER2 expressions were correlated in ovarian cancer, patients were divided into high expression of FASH (high)/HER2 (high) and FASN (high)/HER2 (Low) group (Fig. 4). There was worse overall survival in FASH (high)/HER2 (high) group (*p* = 0.002) (Fig. 5).

## Discussion

Abnormal cell growth is one of the characteristics of cancer cells; the rapid growth of cells requires energy metabolism support [7]. The present study focuses on the correlation between ovarian cancer and expressions of FASN and HER2. FASN is the key enzyme involved in the regulation of cellular fatty acid synthesis and is highly expressed in many cancers such as lung cancer, breast cancer, prostate cancer, colon cancer and many other tumors [8–11]. In the study, we found that FASN has abnormally high expression in ovarian cancer, which was consistent with previous report [12]. High stage of cancer cells often has faster proliferative capacity. A significantly increase in FASN expression in high stage of ovarian cancer was presented, which could be due to the fact that overexpression of FASN can provide material and energy for the rapid proliferation of tumor. Gansler TS deemed that FASN was an independent prognostic indicator for tumor [12]. The present results revealed that the intensity of FASN expression and survival rate of ovarian cancer patients were closely linked, although further study is necessitated to ascertain the critical role of FASN as a prognostic marker for ovarian cancer.

HER2 is a proto-oncogene that controls cell proliferation [4]; there is a large difference in the expression of HER2 in ovarian cancer, the overall scope of the present report is 5–30 % [13–16]. In this research, we outlined that the overall expression of HER2 in ovarian carcinoma was about 33 %. Most of the literature suggests HER2 protein overexpression is associated with poor prognosis in ovarian cancer with a single factor analysis. This was reflected that HER2 overexpression is correlated with increased risk of death, shortened disease progression-free survival and overall survival rate in patients [17, 18]. Nevertheless, the results of multivariate analysis are still controversial. Camilleri-Broet believed that HER2 protein expression is the independent prognostic factors via multivariate analysis [19]. However, Riener EK reported that HER2 overexpression is not an independent prognostic factor for ovarian cancer, and its role for predicting disease needs to rest on other factors, such as, clinical stage, histological grade and residual tumor volume [16]. We found that there was positive rate increased in high stage of ovarian cancer. The patients with high expression of HER2 tended to acquire worse survival rate. The difference was found to be no statistically significant. This suggests that HER2 may be able to influence the prognosis of patients. These findings should be interpreted with caution. Resulting in the variety of HER2 expression and the differences may be associated with different affinity of antibody, clinical pathology laboratory, racial, tumor staging, evaluation standard of HER2 positive evaluation standard and sample. HER2 is likely to become an independent risk factor for prognosis; further evaluations are required in the near future.

Some literature for ovarian cancer suggested that FASN could regulate the expression of HER2 through PI3k-Akt pathway [20]. Similarly, HER2 can regulate the expression of FASN through the reverse pathway [21]. This suggested that FASN and HER2 might have a mutual role in triggering the tumor. The present study suggested no obvious correlation between FASN and HER2 expressions, and this might be due to the relatively small size of samples. However, we found simultaneously that the 5-year survival rate in patients with FASN and HER2 expressions was comparatively lower than that in patients with a single expression. Co-expression of FASN and HER2 might be a sign of poor prognosis tumor.

In the present study, we establish an association between the prognosis of patients with ovarian cancer and FASN/HER2 expressions in ovarian tissue. FASN and HER2 expressions might be associated with prognosis in malignant ovarian cancer.

## Conclusions

We firstly put the first evidence that a cross talk in FASN and HER2 expressions might be associated with prognosis in malignant ovarian cancer.
